# Comparative genomics reveals multiple pathways to mutualism for tick-borne pathogens

**DOI:** 10.1186/s12864-016-2744-9

**Published:** 2016-07-02

**Authors:** Svetlana Lockwood, Kelly A. Brayton, Shira L. Broschat

**Affiliations:** School of Electrical Engineering and Computer Science, Washington State University, P.O. Box 642752, Pullman, USA; Department of Veterinary Microbiology and Pathology, Washington State University, P.O. Box 647040, Pullman, 99164-7040 USA; Paul G. Allen School for Global Animal Health, Washington State University, PO Box 647090, Pullman, 99164-7090 USA

**Keywords:** Tick-borne pathogens, Phylogenomic network, Comparative genomics

## Abstract

**Background:**

Multiple important human and livestock pathogens employ ticks as their primary host vectors. It is not currently known whether this means of infecting a host arose once or many times during evolution.

**Results:**

In order to address this question, we conducted a comparative genomics analysis on a set of bacterial pathogens from seven genera – *Borrelia, Rickettsia, Anaplasma, Ehrlichia, Francisella, Coxiella*, and *Bartonella*, including species from three different host vectors – ticks, lice, and fleas. The final set of 102 genomes used in the study encoded a total of 120,046 protein sequences. We found that no genes or metabolic pathways were present in all tick-borne bacteria. However, we found some genes and pathways were present in subsets of tick-transmitted organisms while absent from bacteria transmitted by lice or fleas.

**Conclusion:**

Our analysis suggests that the ability of pathogens to be transmitted by ticks arose multiple times over the course of evolution. To our knowledge, this is the most comprehensive study of tick transmissibility to date.

**Electronic supplementary material:**

The online version of this article (doi:10.1186/s12864-016-2744-9) contains supplementary material, which is available to authorized users.

## Background

Based on World Health Organization estimates, in recent years the confounding factors of climate change and human demographics have had a significant impact on the increase in vector-borne diseases [1]. A number of severe diseases are caused by different vector-borne bacteria. According to the Centers for Disease Control and Prevention, *Borrelia burgdorferi*, a member of the order *Spirochaetes*, infects 300,000 people with Lyme disease in the United States annually [2]. Bites by ticks carrying the highly infectious bacterium *Francisella tularensis* result in the potentially fatal disease tularemia [3, 4], and more than 20 types of fever and typhus worldwide are attributed to tick-borne members of the spotted fever group of *Rickettsia* [5].

Vector-borne pathogens exhibit remarkable evolutionary adaptations, which allow them to exploit an array of vector hosts even within one genus. *Borrelia hermsii*, the agent of tick-borne relapsing fever, uses as its vector soft-bodied ticks of the genus *Ornithodoros* [6] while *Borrelia burgdorferi*, the causative agent of Lyme disease, is transmitted by the hard-bodied tick *Ixodes scapularis* [7]. Interestingly, these host ticks are widely divergent, separated by millions of years of evolution [8]. Fleas are well-established vectors for *Bartonella henselae* [9–12] while *Bartonella quintana* utilizes lice [13]. Even more remarkably, members of the genus *Rickettsia* use a range of different vectors – fleas (*R. typhi, R. felis*), lice (*R. prowazekii*), mites (*R. akari*), and ticks (*R. parkeri, R. japonica, R. helvetica*, and others).

Among pathogen vectors, ticks are of particular concern as they play a prominent role in transmission of pathogens to humans and livestock. In the US, for example, incidents of Lyme disease almost tripled in the past two decades resulting in $0.7–$1.3 billion in direct medical costs [14–17]. In Tanzania, economic losses resulting not only from direct production losses, but also from treatment and control costs associated with tick-borne diseases (TBDs) alone, are estimated to be 364 million USD annually [18]. Given the significant impact of TBDs both on human health and on economic activities, it is important to understand the biological mechanisms of pathogen transmissibility. However, bio-molecular determinants of tick-borne transmission are still largely unknown.

An intriguing question is whether the ability for a bacterial pathogen to be tick transmitted evolved one or more times. We hypothesized that there may exist a common molecular mechanism among all tick-associated bacterial pathogens. Identification of such a mechanism across different species would suggest a path for the development of transmission blocking strategies. We test our hypothesis by undertaking a comparative genomics approach to analyze bacteria in the genera *Borrelia, Rickettsia, Anaplasma, Ehrlichia, Francisella, Coxiella*, and *Bartonella*, conducting the most comprehensive study examining tick transmissibility to-date.

## Methods

To explore the bio-molecular evidence of tick transmissibility we collected data for all vector-borne bacteria for which full genome sequences are available and a vector is known. These included organisms from seven genera – *Borrelia, Bartonella, Rickettsia, Anaplasma, Ehrlichia, Coxiella*, and *Francisella* (Table 1). From these microorganisms a set was selected for the most prevalent arthropod vectors – ticks, fleas, and lice. Genomes for *R. akari, Orienta tsutsugamushi*, and *Neorickettsia sennetsu* were excluded from the analysis due to the lack of sufficient representation of their particular vectors – chiggers, mites, and trematodes, respectively. We did not include species for which vectors were either unconfirmed or unknown. In addition, a few *Rickettsia* genomes and one *Borrelia* genome were excluded because annotation for them does not exist in the KEGG organism pathway database. The final set comprised 102 genomes with a total of 120,046 protein sequences (Table 1, Additional file 1: Table S1). All complete genomes (plasmids were not used in this study) were downloaded via NCBI ftp service (ftp://ftp.ncbi.nlm.nih.gov/genomes) in August 2015. KEGG databases (http://www.genome.jp/kegg/) were accessed in October 2015 [19, 20].

**Table 1:** Numbers of genomes examined and their vector association

Genus	Vectors			Number of genomes
	Tick	Louse	Flea	
*Anaplasma*	6	0	0	**6**
*Bartonella*	0	2	4	**6**
*Borrelia*	17	1	0	**18**
*Coxiella*	5	0	0	**5**
*Ehrlichia*	14	0	0	**14**
*Francisella*	13	0	0	**13**
*Rickettsia*	26	10	4	**40**
Total	**81**	**13**	**8**	**102**

Boldface used for emphasis

**Fig. 1:**
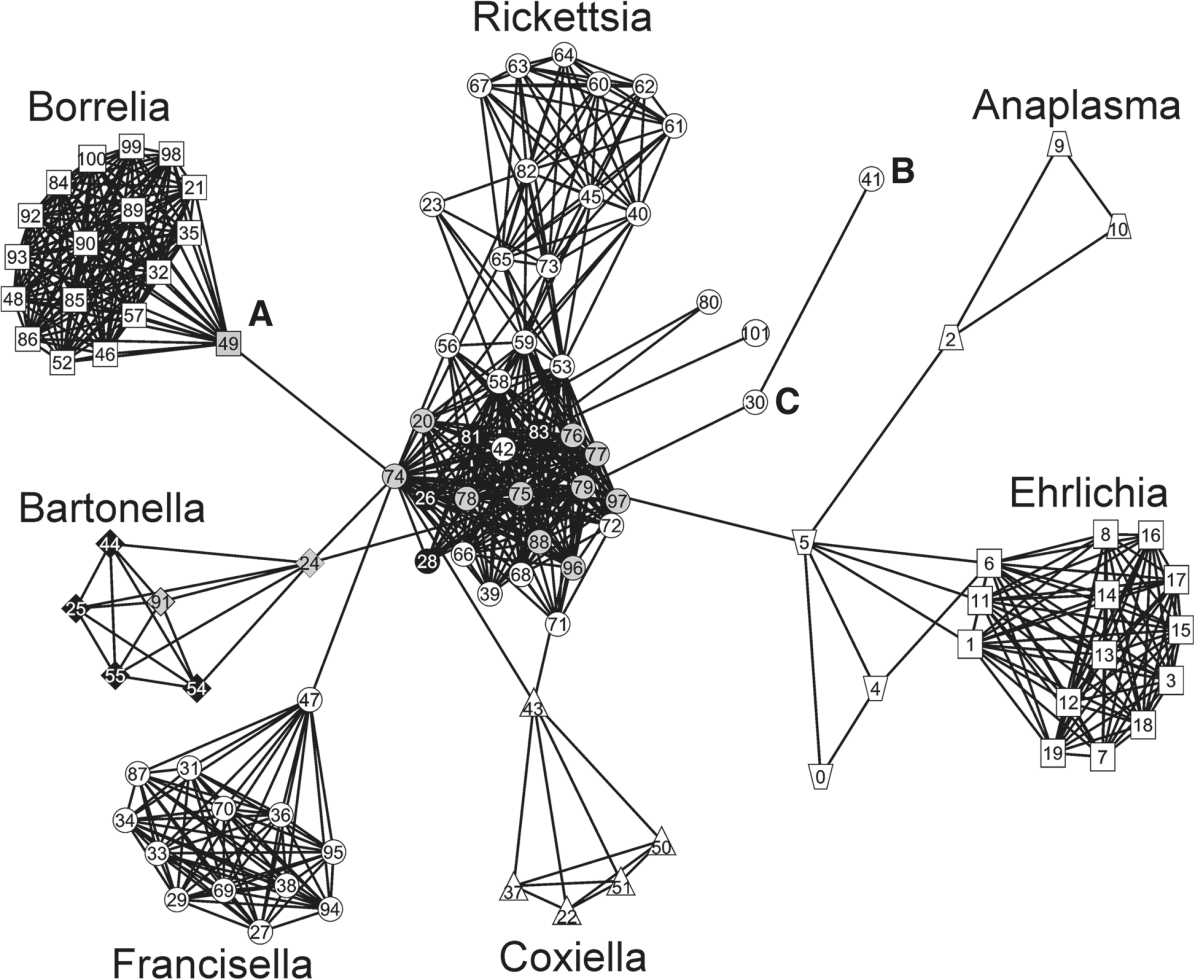
Phylogenomic network of seven genera of arthropod transmitted bacterial pathogens. A node represents an organism, and a link exists between organisms when they possess a certain level of similarity. Shapes for different species – *squares* for *Borrelia* spp. (*top left*) and *Ehrlichia* spp. (*right*), *diamonds* for *Bartonella* spp. (*left*), *triangles* for *Coxiella* spp. (*bottom*), *trapezoids* for *Anaplasma* spp. (*top and near right*), *circles* for *Francisella* spp. (*bottom left*) and *Rickettsia* spp. (*center and top*). Shading for different vectors – *black* for fleas, *gray* for lice, *white* for ticks. Letters outside the nodes mark the following microorganisms: *A* – *Borrelia recurrentis* A1, *B* – *Rickettsia bellii* OSU 85-389, *C* – *Rickettsia bellii* RML369-C. Numbers inside the nodes correspond to the organism number column in Additional file 1: Table S1

A word on the notation used in the consecutive sections of this article. The pound sign and number following an organism name refer to the microorganism number in supplementary Table S1 (Additional file 1) and the number in the network in Fig. 1. For example, *R. canadensis* str. McKiel (#39) indicates that the organism number is 39 both in the network of Fig. 1 and in Table S1 (Additional file 1).

The open-source GUI software package *pClust* and custom R scripts were used for phylogenomic network construction. *pClust* utilizes the *Parasail* package for fast sequence alignment [21, 22] and *Grappolo* for clustering [23]. Network visualization was done using the free software package *visone* [24]. Sequence alignment and clustering by means of *pClust* was performed on a desktop machine running Windows 8 with 8 GB of RAM and took less than 10 minutes for the 102 genomes. For more details and specific parameters for phylogenomic network construction and visualization as well as for information on downloading the software, consult Additional file 2.

## Results

### Genome clustering

Employing the *pClust* clustering procedure (Additional file 2), we found distinct cluster separation among different vector-borne organisms (Fig. 1). Overall, the network structure appears to be driven by divisions among different genera, but some important insights into relationships among the species are also provided. The tick-borne (white symbols) *Borrelia* (Fig. 1, squares top left), *Francisella* (Fig. 1, circles bottom left), and *Ehrlichia* (Fig. 1, squares far right) create clearly defined clusters with thicker meshes indicating closely related organisms. Within the *Bartonella* cluster (Fig. 1, diamonds left), a flea-borne (black symbols) group of bacteria is clearly defined (black diamonds). Tick-borne *Anaplasma phagocytophilum* forms a triangle (Fig. 1, trapezoids top right) independent from *Ehrlichia* while the *A. marginale* and *A. centrale* triangle (Fig. 1, trapezoids near right) is much closer to the *Ehrlichia* spp.

The order *Rickettsiales* was reorganized based on phylogenetic data from a few genes [25]. Our study is based on whole genome analysis. The genera *Anaplasma* and *Ehrlichia* have always been recognized as being more closely related to each other than to the other genera (*Wolbachia* and *Neorickettsia*) in the family. *A. centrale* is a subspecies of *A. marginale*, and therefore very closely related, so it makes sense that these would cluster very tightly. *A. phagocytophilum* has a larger genome than the other *Anaplasma* species and has an expansion of the *msp2* superfamily. In addition, it contains more Ank domain effector molecules. These differences may be sufficient to set its genome/proteome apart given the very limited set of complete genomes examined. For example, there are no complete genomes available for *A. platys* or *A. ovis*, which, if available, might provide a slightly different picture of how these two genera cluster.

The *Rickettsia* cluster (Fig. 1, circles center and top) is not as compact as other clusters and several subgroups are distinguishable within it. Notably, it consists of the tick-borne *R. rickettsii* at the top, but the central core group contains in its center louse-borne (gray circles) and flea-borne (black circles) *Rickettsia*. It is interesting to note that *Borrelia recurrentis* (Fig. 1, A), a louse-transmitted species, which has a genome that appears to be a degraded subset of the tick-transmitted *B. duttonii* [26], serves as a transitional organism between the major body of the *Rickettsia* and *Borrelia* clusters. Also noteworthy is the independent positions of the *R. bellii* organisms (Fig. 1, B and C) which, together with *R. canadensis* (#39 and #66), are considered to form the ancestral group of *Rickettsia* [27]. While the organism clustering is genus-driven, it is intriguing that clusters emanating from the *Rickettsia* group are connected by their transitional organisms to a louse-borne species in the center of the *Rickettsia* cluster, i.e., the *Bartonella, Borrelia, Francisella, Coxiella*, and *Ehrlichia* (via *Anaplasma* spp.) clusters are connected to the *Rickettsia* cluster via louse-borne organisms (Fig. 1).

The origins and the ability of pathogens to be insect-borne are also still largely unknown. Current evidence suggests that the genomes of louse-borne organisms are the result of degraded genomes from their arthropod-borne relatives. For example, *Borrelia duttonii*, which is tick-borne, degraded to *B. recurrentis* which is louse-borne with 20 % genome reduction [26]; *Bartonella henselae*, a flea-borne pathogen, degraded to louse-borne *B. quintana* with 12.6 % genome reduction [28]; and *Rickettsia conorii* (tick-borne) degraded to *R. prowazekii* (louse-borne) with 18 % genome reduction [29]. Both louse- and flea-borne organisms are positioned in the center of the *Rickettsia* spp. cluster (Fig. 1), and many transitional genomes are connected to them. For example, *Borrelia recurrentis* (#49), a louse-borne transitional organism for *Borrelia* spp., is connected to the louse-borne *R. prowazekii* str. GvV257 (#74). *Bartonella quintana* str. Toulouse (#24), a louse-borne transitional organism for *Bartonella* spp., and *C. burnetii* RSA 331 (#43), a tick-borne transitional organism for *Coxiella* spp., are both connected to two louse-borne *R. prowazekii* strains – str. GvV257 (#74) and str. RpGvF24 (#79). At the same time the tick-borne *A. centrale* str. Israel (#5), a transitional organism for *Anaplasma* spp., is connected only to the louse-borne *R. prowazekii* str. RpGvF24 (#79).

### No genes are uniquely present in all tick-borne bacteria

**Fig. 2:**
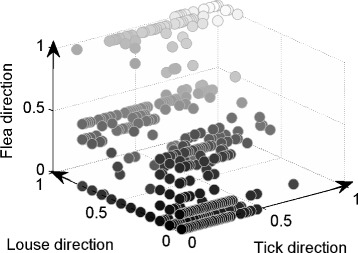
Three-dimensional depiction of gene cluster representation according to vector species. Every ball represents a gene cluster, and its location in (*x, y, z*) coordinates gives the fraction or percentage proportional to its appearance in the genome of an organism that is transmitted by one of the three vector types – ticks, lice, and fleas, respectively

If a set of genes common to all tick-borne bacteria exists, then it implies there is a bio-molecular, tick-associated mechanism shared among all of them. Note that while we refer to homologous gene (HG) clusters, we actually clustered deduced amino acid sequences, or proteins; we use the terms synonymously. We examined the microbial genomes for homologous gene presence using 3D visualization (Fig. 2, Additional file 3: Figure S1). In Fig. 2, every ball represents a cluster of homologous genes, and its location in (*x, y, z*) coordinates gives the fraction or percentage proportional to its appearance in the genome of an organism that is transmitted by one of the three vector types – ticks, lice, and fleas, respectively. For example, if a certain cluster is located at coordinates (0.4,0.3,0.7), it means that the cluster appears in 40 % of tick-borne organisms, 30 % of louse-borne organisms, and 70 % of flea-borne organisms in our set. Such a mapping allows quick identification of vector-specific clusters. For example, all homologous gene clusters unique only to tick-borne organisms would have coordinates (*x*,0,0), or, in other words, would lie on the tick-axis. Similarly, homologous gene clusters present exclusively in louse- or flea-borne organisms would lie on the louse-axis and flea-axis, respectively. Homologous genes present in all organisms are located at (1,1,1).

**Fig. 3:**
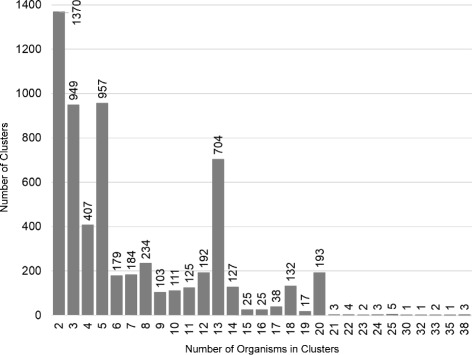
Multiplicity plot of homologous gene clusters along the tick-axis. The bars represent the number of gene/protein clusters that are comprised of the number of tick-transmitted species represented on the *x*-axis, e.g., the rightmost bar indicates that there are 38 tick transmissible species that have three clusters in common (also see Additional file 3: Figure S2)

In Fig. 2 we observe that no gene cluster is at coordinates (1,0,0), which indicates that there are no homologous genes present exclusively and entirely in all tick-borne organisms and, thus, no single set of genes is responsible for all tick-borne transmission. The same is true for both louse- and flea-borne organisms, although our sampling of these genomes is smaller. However, there are sets of homologous gene clusters that are unique to various subsets of tick-borne bacteria. To understand this, we constructed a multiplicity 3D plot in which the size of each ball indicates the number of the homologous gene clusters at that point (Additional file 3: Figure S1). For example, clusters that are present in few organisms are located closer to zero and they have high multiplicity as indicated by their sizes, i.e., balls are large. Because we are interested in tick transmissibility, we examine only the clusters located along the tick-axis. Figure 3 shows the multiplicity of each gene cluster along the tick-axis. For example, the last ball on the tick-axis (Additional file 3: Figure S1) corresponds to the last column in Fig. 3, from which we see that there are three different HG clusters present in 38 tick-borne organisms. Figure S2 (Additional file 3) shows exactly how the three clusters span the tick-borne organisms. In this case, the three HG clusters are present in closely related *Anaplasma* and *Ehrlichia* spp., but also in the more distant species of *Francisella* and *Coxiella* (Additional file 2: Figure S2). It is quite remarkable that organisms as different as *Anaplasma* and *Francisella* have common genes that are not present in any of the louse- or flea-borne organisms. A similar situation is shown in Figure S3 (Additional file 3) in which two HG clusters span three genera of tick-borne organisms – *Anaplasma, Ehrlichia*, and *Francisella*.

### There are pathways present in sets of tick-borne bacteria that are absent from bacteria transmitted by lice or fleas

We systematically examined various metabolic pathways associated with the tick-borne HG clusters spanning several genera. In particular, we examined all pathways associated with the last ten homologous gene clusters present in significant numbers in only tick-borne microorganisms. Table 2 details information for 25 unique HG clusters. While we did not find differences with respect to complete metabolic pathways, we did find several subpaths that are present in some genera while absent in others. In particular, we found that *Anaplasma, Ehrlichia, Francisella*, and *Coxiella* spp. all share parts of major metabolic pathways that are absent in any louse- or flea-borne bacteria.

**Table 2:** Detailed information for the most widely present homologous gene clusters

Cluster #	Representative protein accession #	# of organisms in cluster	Product description	KEGG EC #
30	AAV86731	38	2,3-bisphosphoglycerate-independent phosphoglycerol mutase	5.4.2.12
30	AAV87091	38	nicotinate-nucleotide pyrophosphorylase	2.4.2.19
30	AAV87100	38	quinolinate synthetase A	2.5.1.72
29	NP_212375	35	glycerol kinase	2.7.1.30
28	AAV86691	33	1-deoxy-D-xylulose-5-phosphate reductoisomerase	1.1.1.267
28	AAV87137	33	2-C-methyl-D-erythritol 2,4-cyclodiphosphate synthase	4.6.1.12
27	AHX03201	32	tRNA threonylcarbamoyladenosine biosynthesis protein TsaE	no EC number
26	NP_212377	30	glycerol-3-phosphate dehydrogenase	1.1.5.3
25	AAV87130	25	hypothetical	no entry
25	NP_359873	25	hypothetical	no entry
25	AAV86484	25	pyridoxine 5-phosphate synthase	2.6.99.2
25	AAV86592	25	bifunctional proline dehydrogenase, pyrroline-5-carboxylate dehydrogenase	1.5.5.2, 1.2.1.88
25	AAV86778	25	thiamine biosynthesis oxidoreductase	1.4.3.19
24	NP_359861	24	divalent cation tolerance protein	no entry
24	NP_360910	24	hypothetical	no entry
24	NP_360096	24	hypothetical	no entry
23	NP_360134	23	hypothetical	no entry
23	NP_360791	23	hypothetical	no entry
22	YP_001499275	22	AbrB family transcriptional regulator	no entry
22	NP_360166	22	cell filamentation protein Fic^a^	no entry
22	NP_360392	22	hypothetical	no entry
22	NP_360955	22	plasmid stability protein	no entry
21	NP_359844	21	hypothetical	no entry
21	NP_360418	21	biotin-protein ligase	no entry
21	NP_360956	21	hypothetical	no entry

^a^This gene was reported as one of three representative ORGs, RiOG_1005, i.e., present in all tick-borne *Rickettsial* genomes in the study by Gillespie *et al*. [27]

**Fig. 4:**
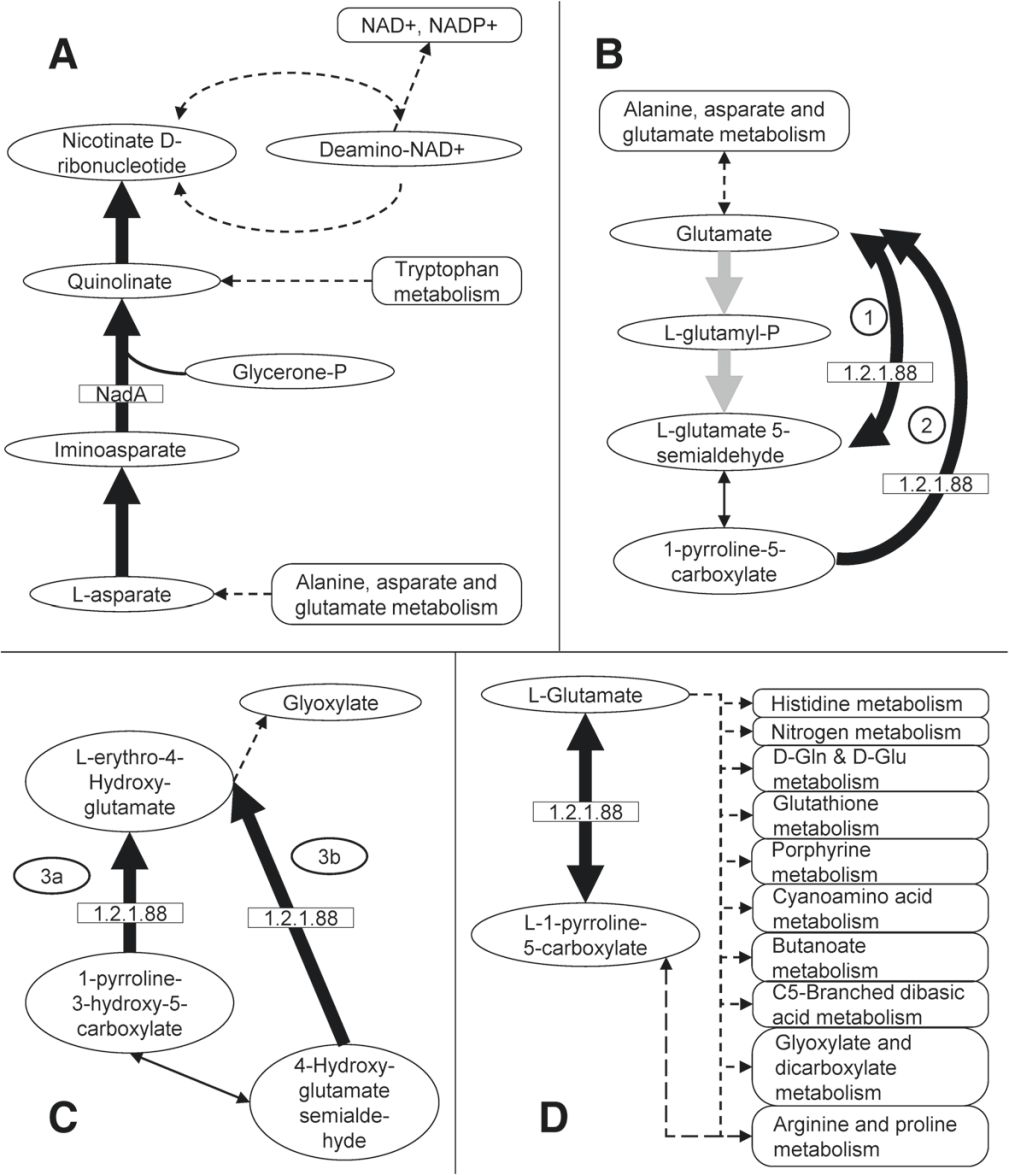
Pathway analysis for different organisms. *Thick black or gray arrows* – enzyme-dependent subpaths; *thin black arrows* – enzyme-independent conversions; *dashed arrows* – other metabolic paths. *Ovals* – intermediate products; *rectangles* – enzymes of interest; *rounded rectangles* – major metabolic paths. **a** Part of nicotinate and nicotinamide metabolism pathway (KEGG:ko00760). This subpath is present in *Anaplasma, Ehrlichia, Francisella*, and *Coxiella* spp. In *Francisella* and *Coxiella* – from L-asparate to nicotinate D-ribonucleotide. In *Anaplasma* and *Ehrlichia* – from glycerone-P to nicotinate D-ribonucleotide. The path is absent in *Bartonella, Rickettsia*, and *Borrelia* spp. **b**, **c** Two subpaths of arginine and proline metabolism pathways (KEGG:ko00330). Both are present in *Anaplasma, Ehrlichia, Francisella*, and *Coxiella* spp. while absent in *Rickettsia, Borrelia*, and *Bartonella* spp., which can complete only the *gray arrow* path in B. **d** Part of alanine, aspartate, and glutamate metabolism pathway (KEGG:ko00250). Present in *Anaplasma, Ehrlichia, Francisella*, and *Coxiella* spp. while absent in *Rickettsia, Borrelia*, and *Bartonella* spp

Table 3 summarizes this information. The first example is a part of the nicotinate and nicotinamide metabolism pathway from L-asparate to nicotinate D-ribonucleotide (Table 3, Fig. 4a). Nicotinate and nicotinamide are the precursors for generation of coenzymes NAD+ and NADP+ [30, 31]. These coenzymes are crucial for many metabolic pathways including glycolysis, TCA cycle, pentose phosphate cycle, fatty acid biosynthesis and metabolism pathways, and many others [30, 31]. The path from L-asparate to nicotinate D-ribonucleotide is present in *Anaplasma, Ehrlichia, Francisella*, and *Coxiella* spp. – all tick-borne species (Fig. 4a). In *Francisella* and *Coxiella* this part of the pathway starts at L-asparate while in *Anaplasma* and *Ehrlichia* it starts from the freely available glycerone-P. The subpath is absent from *Rickettsia* and *Borrelia* spp., although some of these are tick-borne, as well as from *Bartonella* spp., which has only flea- and louse-borne organisms.

**Table 3:** Examples of pathways present in a subset of tick-borne genera

KEGG KO #and pathway	Product	Tick-borne				Louse-borne				Flea-borne		
		A^a^	E	F	C	Bo	R	R	Bo	Ba	Ba	R
00760: Nicotinate and nicotinamide metabolism	nicotinate D-ribonucleotide	+	+	+	+	-	-	-	-	-	-	-
00250: Alanine, aspartate and glutamate metabolism	various	+	+	+	+	-	-	-	-	-	-	-
00330: Arginine and proline metabolism	various	+	+	+	+	-	-	-	-	-	-	-

^a^Genus abbreviations: A=*Anaplasma*; E=*Ehrlichia*; F=*Francisella*; C=*Coxiella*; Bo=*Borrelia*; Ba=*Bartonella*; R=*Rickettsia*

Another example is subpaths in the arginine and proline metabolism pathway (Fig. 4b–c). In this major pathway, bifunctional enzyme 1-pyrroline-5-carboxylate dehydrogenase (KEGG:EC 1.2.1.88) is responsible for a number of chemical reactions including 1) reversal from glutamate to L-glutamate 5-semialdehyde and back (Fig. 4b), 2) from 1-pyrroline-5-carboxylate to glutamate (Fig. 4b), and 3) from 1-pyrroline-3-hydroxy-5-carboxylate (Fig. 4c, path 3a) or 4-hydroxyglutamate semialdehyde (Fig. 4c, path 3b) to L-erythro-4-hydroxy-glutamate which further gets transformed into glyoxylate or pyruvate. These subpaths are present in *Anaplasma, Ehrlichia, Francisella*, and *Coxiella* spp. Neither *Rickettsia* nor *Borrelia* spp. can complete any of these while *Bartonella* spp. can only complete one-way conversion of subpath 1, i.e., from glutamate to L-glutamate 5-semialdehyde, but not back (Fig. 4b, gray arrows).

The absence of bifunctional 1-pyrroline-5-carboxylate dehydrogenase in *Rickettsia, Borrelia*, and *Bartonella* spp. causes disruption in a part of another major amino acid metabolic pathway, namely in the alanine, aspartate, and glutamate metabolism pathway (Fig. 4d). The pathway part – reversible conversion from L-1-pyrroline-5-carboxylate to L-glutamate – is present in *Anaplasma, Ehrlichia, Francisella*, and *Coxiella* spp. while *Rickettsia, Bartonella*, and *Borrelia* spp. do not have this subpath.

## Discussion

We hypothesized that the ability of pathogens to be tick transmitted arose once in evolution. If this is correct, then we would expect to find a set of genes common to all tick-borne bacteria and, presumably, these genes would confer tick transmissibility. To test this hypothesis, we selected a wide variety of vector-borne bacteria including species from multiple genera as well as non-tick-borne bacteria for contrast. To emphasize tick association, we looked at the set of homologous genes (HGs) that are present only in tick-borne pathogens and absent in flea- and louse-borne bacteria. We found that there are no tick-associated genes that are present in all tick-borne bacteria.

The absence of HGs unique to all tick-borne pathogens suggests their ability to be tick transmitted arose multiple times in evolution. An alternative possibility is that genes involved in the ability to be tick transmitted accumulated mutations to the extent that they are no longer recognized as homologs. Another possibility is that the ability of pathogens to be tick transmitted may not require a unique set of genes. However, if this were true, an organism with the basic set of essential genes would be tick transmissible, but this is not the case. Thus, given the absence of homologous genes shared by all tick-borne pathogens, we suggest that the ability of pathogens to be tick transmitted arose multiple times during their evolution, and different sets of organisms employ different sets of genes to confer this ability. We did find homologous genes that are common to different subsets of tick-borne organisms. These subsets consist of different combinations of *Anaplasma, Ehrlichia, Francisella*, and *Coxiella* spp. (Additional file 3: Figures S2, S3). Observe that no cross set of genes was found that included *Rickettsia* and other genera. This suggests parallel evolutionary development of tick association for *Rickettsia* and other species.

The existence of genes unique to tick-borne bacteria among the set of organisms analyzed does not necessarily suggest that the metabolic processes in which these genes are involved cannot be completed by other means, for example, either by employing a different enzyme or by completing a different pathway. However, the tick-specific homologous genes suggest where the tick-specific metabolic pathways might exist. Thus, we considered whether sufficient evidence could be detected for complete metabolic pathways by analyzing the pathways associated with the tick-specific HGs we identified. Because all organisms in our study are vector-borne, many of them have undergone reductive evolution and rely on the host cell environment to supply necessary substrates and chemical compounds [32–34]. Whether a pathway with few enzymes in it can be fully functional or not is unknown. Some experimental evidence exists showing that metabolic reactions can be successfully completed by harvesting necessary substrates from the host environment. For example, Frohlich et al. showed that while *R. prowazekii* is unable to synthesize DHAP as a substrate for the GpsA enzymatic reaction, it encodes and synthesizes a functional GpsA enzyme [35]. Therefore, in our pathway analysis rather than considering completion of a full metabolic pathway, we concentrated on whether or not a certain part, a subpath, of a metabolic pathway could be completed. In this way we found a few metabolic subpaths that are unique to tick-borne bacteria via the HGs associated with them.

Two of the subpaths are part of amino acid metabolic pathways and one is part of the nicotinate and nicotinamide metabolism pathway. Of particular interest is the latter pathway. Nicotinamidase, or PncA, is an enzyme involved in the production of NAD and was empirically shown to play an important role in the infectivity of Borrelia [36]. When a non-infectious, low-passage strain of *B. burgdorferi* was introduced with a copy of a gene coding for nicotinamidase on a shuttle vector, viable *B. burgdorferi* were recovered from all tissue samples [36]. Interestingly, the common subpath we found from L-asparate via quinolinate to nicotinate D-ribonucleotide (Fig. 4a) is present in *Anaplasma, Ehrlichia, Francisella*, and *Coxiella* spp. Thus, these organisms prefer an endogenous pathway for NAD synthesis while *Rickettsia* and *Bartonella* spp. must rely on other means such as, for example, in the case of *Borrelia* spp. which employ exogenous pathways for NAD synthesis [36].

We must note that our pathway analysis is not exhaustive. One reason for this is that functionality of many gene products is unknown and, thus, their participation in metabolic pathways cannot be defined. There may exist other sets of metabolic pathways common to tick- and other vector-borne pathogens, but their identification is hindered due to our limited knowledge. For example, 10 of 25 examined homologous genes do not have annotation in KEGG pathways because they are hypothetical (Table 2). Another reason is that while our knowledge of gene presence is complete – that is, there are only so many genes in a particular organism and, if a genome is sequenced, all of them are known – our current knowledge of metabolic pathways is far from complete. For example, an additional 4 of 25 examined homologous genes have no associated KEGG entry although their products are known (Table 2). This fact reflects the inherent complexity associated with biochemical processes in living organisms and our ongoing efforts to understand them.

It is interesting to note that just as there are no HGs common to all tick-borne pathogens, there are no HGs common to all louse- and flea-borne organisms as well. It is quite a remarkable fact given the relatively small set of organisms associated with these vectors examined in the present study. This is because the likelihood of organisms sharing common genes decreases as the size and diversity of the set grow. In the current set of organisms there are only 8 flea- and 13 louse-associated pathogens. Along the flea-axis (Fig. 2), the farthest point is located at (0,0,0.5), which has multiplicity 20 meaning that 50 %, or equivalently 4 out of 8, flea-borne organisms share 20 HGs. Close examination shows they are present only in flea-borne members of *Bartonella* (data not shown). A similar situation exists for louse-borne organisms. Along the louse-axis the farthest point is located at (0,0.77,0), which has multiplicity 5 meaning that 77 %, or equivalently 10 out of 13 louse-borne organisms share 5 HGs and, again, all of them are present in only one genus, namely *Rickettsia*. Thus, in the case of louse- and flea-borne bacteria there are no genes that span multiple genera unlike the situation for tick-borne pathogens. In part this may be explained by the fact that we used only two genera with flea-borne organisms – *Bartonella* and *Rickettsia* – and three genera with louse-borne organisms – *Borrelia, Bartonella*, and *Rickettsia*. Future research may address this shortcoming.

## Conclusion

We initiated our study with the hypothesis that there may exist a common molecular mechanism among all tick-associated pathogens. Such a common mechanism would greatly benefit our understanding of the pathogenicity of tick-borne microbial organisms. However, our analysis indicates that rather than having one molecular mechanism common to all tick-borne bacteria, there are several evolutionary paths that, perhaps, developed independently as different organisms spread into new hosts and acquired new niches. We also found a number of hypothetical gene products common to a significant number of organisms in our set. A tantalizing question remains open whether these genes hold the clues for molecular mechanisms specific to lifestyles within the arthropod host.

## Abbreviations

DHAP, dihydroxyacetone phosphate; GUI, graphical user interface; HG, homologous gene; KEGG, Kyoto Encyclopedia of Genes and Genomes; MB, megabyte; NAD, nicotinamide adenine dinucleotide; NADP, nicotinamide adenine dinucleotide phosphate; NCBI, national center for biotechnology information; RAM, random access memory; TBD, tick-borne disease.

## Additional files

Additional file 1 Organisms and their protein sequences. **Table S1.** Organisms and their protein sequence files in the current study. (PDF 385 kb)

Additional file 2 Network construction. Network construction methodology and baseline comparison study for network construction. (PDF 820 kb)

Additional file 3
**Figure S2.** The span of three gene clusters across different tick-borne organisms. **Figure S3.** The span of two gene clusters across different tick-borne organisms. (PDF 606 kb)
