# Supplementary material for: Comparative genomics reveals multiple pathways to mutualism for tick-borne pathogens
Source: BMC Genomics. 2016 Jul 2;17:481. doi: 10.1186/s12864-016-2744-9 (PMC4930560; doi:10.1186/s12864-016-2744-9)
Supplement: Additional file 1 — Organisms and their protein sequences. Table S1. Organisms and their protein sequence files in the current study. (PDF 385 kb) [file 12864_2016_2744_MOESM1_ESM.pdf]

Table S1. Organisms and their protein sequence files in the current study.

Organism Number[2]	Species	Protein Sequences File	Number of Proteins	Vector
5	*Anaplasma centrale* str. Israel	GCA_000024505.1_ASM2450v1_protein.faa	925	Tick
4	*Anaplasma marginale* str. Florida	GCA_000020305.1_ASM2030v1_protein.faa	942	Tick
0	*Anaplasma marginale* str. St. Maries	GCA_000011945.1_ASM1194v1_protein.faa	949	Tick
2	*Anaplasma phagocytophilum* str. HZ	GCA_000013125.1_ASM1312v1_protein.faa	1352	Tick
9	*Anaplasma phagocytophilum* str. HZ2	GCA_000439755.1_ASM43975v1_protein.faa	1247	Tick
10	*Anaplasma phagocytophilum* str. JM	GCA_000439775.1_ASM43977v1_protein.faa	1256	Tick
55	*Bartonella clarridgeiae* 73	NC_014932.faa	1323	Fleas
54	*Bartonella grahamii* as4aup	NC_012846.faa	1737	Fleas
25	*Bartonella henselae* str. Houston-1	NC_005956.faa	1488	Fleas
91	*Bartonella quintana* RM-11	NC_018533.faa	1203	Lice
24	*Bartonella quintana* str. Toulouse	NC_005955.faa	1142	Lice
44	*Bartonella tribocorum* CIP 105476	NC_010161.faa	2069	Fleas
93	*Borrelia afzelii* HLJ01	NC_018887.faa	892	Tick
32	*Borrelia afzelii* PKo (FLI)[1]	NC_008277.faa	848	Tick
84	*Borrelia afzelii* PKo (Maryland)[1]	NC_017238.faa	824	Tick
57	*Borrelia bissettii* DN127	NC_015921.faa	816	Tick
21	*Borrelia burgdorferi* B31	NC_001318.faa	797	Tick
98	*Borrelia burgdorferi* CA382	NC_022048.faa	819	Tick
85	*Borrelia burgdorferi* JD1	NC_017403.faa	823	Tick
86	*Borrelia burgdorferi* N40	NC_017418.faa	809	Tick
52	*Borrelia burgdorferi* ZS7	NC_011728.faa	808	Tick
90	*Borrelia crocidurae* str. Achema	NC_017808.faa	864	Tick
48	*Borrelia duttonii* Ly	NC_011229.faa	820	Tick
89	*Borrelia garinii* BgVir	NC_017717.faa	826	Tick
92	*Borrelia garinii* NMJW1	NC_018747.faa	813	Tick
46	*Borrelia hermsii* HS1	NC_010673.faa	819	Tick
99	*Borrelia miyamotoi* LB-2001	NC_022079.faa	808	Tick
49	*Borrelia recurrentis* A1	NC_011244.faa	800	Lice
35	*Borrelia turicatae* 91E135	NC_008710.faa	818	Tick
100	*Borrelia valaisiana* VS116	NZ_ABCY02000001.faa	832	Tick
50	*Coxiella burnetii* CbuG_Q212	NC_011527.faa	1864	Tick

51	*Coxiella burnetii* CbuK_Q154	NC_011528.faa	1898	Tick
37	*Coxiella burnetii* Dugway 5J108-111	NC_009727.faa	1993	Tick
43	*Coxiella burnetii* RSA 331	NC_010117.faa	1930	Tick
22	*Coxiella burnetii* RSA 493	NC_002971.faa	1823	Tick
1	*Ehrlichia canis* str. Jake	GCA_000012565.1_ASM1256v1_protein.faa	925	Tick
3	*Ehrlichia chaffeensis* str. Arkansas	GCA_000013145.1_ASM1314v1_protein.faa	1105	Tick
12	*Ehrlichia chaffeensis* str. Heartland	GCA_000632815.1_ASM63281v1_protein.faa	970	Tick
14	*Ehrlichia chaffeensis* str. Jax	GCA_000632865.1_ASM63286v1_protein.faa	989	Tick
15	*Ehrlichia chaffeensis* str. Liberty	GCA_000632885.1_ASM63288v1_protein.faa	989	Tick
16	*Ehrlichia chaffeensis* str. Osceola	GCA_000632905.1_ASM63290v1_protein.faa	988	Tick
17	*Ehrlichia chaffeensis* str. Saint Vincent	GCA_000632925.1_ASM63292v1_protein.faa	973	Tick
18	*Ehrlichia chaffeensis* str. Wakulla	GCA_000632945.1_ASM63294v1_protein.faa	980	Tick
19	*Ehrlichia chaffeensis* str. West Paces	GCA_000632965.1_ASM63296v1_protein.faa	962	Tick
11	*Ehrlichia muris* AS145	GCA_000508225.1_ASM50822v1_protein.faa	904	Tick
7	*Ehrlichia ruminantium* str. Gardel	GCA_000050405.1_ASM5040v1_protein.faa	950	Tick
6	*Ehrlichia ruminantium* str. Welgevonden	GCA_000026005.1_ASM2600v1_protein.faa	888	Tick
8	*Ehrlichia ruminantium* str. Welgevonden	GCA_000050425.1_ASM5042v1_protein.faa	958	Tick
13	Ehrlichia sp. HF	GCA_000632845.1_ASM63284v1_protein.faa	946	Tick
94	*Francisella tularensis* subsp. holarctica F92	NC_019537.faa	1842	Tick
95	*Francisella tularensis* subsp. holarctica FSC200	NC_019551.faa	1438	Tick
38	*Francisella tularensis* subsp. holarctica FTNF002-00	NC_009749.faa	1581	Tick
29	*Francisella tularensis* subsp. holarctica LVS	NC_007880.faa	1754	Tick
33	*Francisella tularensis* subsp. holarctica OSU18	NC_008369.faa	1555	Tick
47	*Francisella tularensis* subsp. mediasiatica FSC147	NC_010677.faa	1406	Tick
34	*Francisella tularensis* subsp. novicida U112	NC_008601.faa	1719	Tick
31	*Francisella tularensis* subsp. tularensis FSC198	NC_008245.faa	1605	Tick
87	*Francisella tularensis* subsp.	NC_017453.faa	1836	Tick

	tularensis NE061598			
27	*Francisella tularensis* subsp. tularensis SCHU S4	NC_006570.faa	1556	Tick
70	*Francisella tularensis* subsp. tularensis TI0902	NC_016937.faa	1544	Tick
69	*Francisella tularensis* subsp. tularensis TIGB03	NC_016933.faa	1624	Tick
36	*Francisella tularensis* subsp. tularensis WY96-3418	NC_009257.faa	1634	Tick
53	*Rickettsia africae* ESF-5	NC_012633.faa	1030	Tick
80	*Rickettsia australis* str. Cutlack	NC_017058.faa	1239	Tick
41	*Rickettsia bellii* OSU 85-389	NC_009883.faa	1475	Tick
30	*Rickettsia bellii* RML369-C	NC_007940.faa	1429	Tick
66	*Rickettsia canadensis* str. CA410	NC_016929.faa	1016	Tick
39	*Rickettsia canadensis* str. McKiel	NC_009879.faa	1089	Tick
23	*Rickettsia conorii* str. Malish 7	NC_003103.faa	1374	Tick
28	*Rickettsia felis* URRWXCal2	NC_007109.faa	1400	Fleas
56	*Rickettsia heilongjiangensis* 054	NC_015866.faa	1297	Tick
58	*Rickettsia japonica* YH	NC_016050.faa	971	Tick
42	*Rickettsia massiliae* MTU5	NC_009900.faa	968	Tick
68	*Rickettsia massiliae* str. AZT80	NC_016931.faa	1195	Tick
101	*Rickettsia monacensis*	NZ_LN794217.faa	1443	Tick
72	*Rickettsia montanensis* str. OSU 85-930	NC_017043.faa	1217	Tick
73	*Rickettsia parkeri* str. Portsmouth	NC_017044.faa	1318	Tick
67	*Rickettsia philipii* str. 364D	NC_016930.faa	1344	Tick
97	*Rickettsia prowazekii* str. Breinl	NC_020993.faa	920	Lice
78	*Rickettsia prowazekii* str. BuV67-CWPP	NC_017056.faa	843	Lice
75	*Rickettsia prowazekii* str. Chernikova	NC_017049.faa	845	Lice
77	*Rickettsia prowazekii* str. Dachau	NC_017051.faa	839	Lice
74	*Rickettsia prowazekii* str. GvV257	NC_017048.faa	829	Lice
76	*Rickettsia prowazekii* str. Katsinyian	NC_017050.faa	844	Lice
20	*Rickettsia prowazekii* str. Madrid E	NC_000963.faa	843	Lice

96	*Rickettsia prowazekii* str. NMRC Madrid E	NC_020992.faa	938	Lice
88	*Rickettsia prowazekii* str. Rp22	NC_017560.faa	950	Lice
79	*Rickettsia prowazekii* str. RpGvF24	NC_017057.faa	834	Lice
71	*Rickettsia rhipicephali* str. 3-7-female6-CWPP	NC_017042.faa	1252	Tick
61	*Rickettsia rickettsii* str. Arizona	NC_016909.faa	1343	Tick
63	*Rickettsia rickettsii* str. Brazil	NC_016913.faa	1332	Tick
60	*Rickettsia rickettsii* str. Colombia	NC_016908.faa	1350	Tick
62	*Rickettsia rickettsii* str. Hauke	NC_016911.faa	1340	Tick
64	*Rickettsia rickettsii* str. Hino	NC_016914.faa	1335	Tick
65	*Rickettsia rickettsii* str. Hlp#2	NC_016915.faa	1308	Tick
45	*Rickettsia rickettsii* str. Iowa	NC_010263.faa	1382	Tick
40	*Rickettsia rickettsii* str. 'Sheila Smith'	NC_009882.faa	1343	Tick
59	*Rickettsia slovaca* 13-B	NC_016639.faa	1112	Tick
82	*Rickettsia slovaca* str. D-CWPP	NC_017065.faa	1347	Tick
81	*Rickettsia typhi* str. B9991CWPP	NC_017062.faa	839	Fleas
83	*Rickettsia typhi* str. TH1527	NC_017066.faa	838	Fleas
26	*Rickettsia typhi* str. Wilmington	NC_006142.faa	837	Fleas
	Total: 102 organisms		***120,046***	

[1] *FLI* and *Maryland* are designations in the KEGG database for the two *Borrelia afzelii* spp.

[2] Network number corresponds to the organism number in the network in Figure 1.
