# Supplementary material for: Comparative genomics reveals multiple pathways to mutualism for tick-borne pathogens
Source: BMC Genomics. 2016 Jul 2;17:481. doi: 10.1186/s12864-016-2744-9 (PMC4930560; doi:10.1186/s12864-016-2744-9)
Supplement: Additional file 2 — Network construction. Network construction methodology and baseline comparison study for network construction. (PDF 820 kb) [file 12864_2016_2744_MOESM2_ESM.pdf]

Network Construction

The phylogenomic network shown in Fig. 1 was constructed using a number of sequential steps. First, protein sequences for all organisms in this study were downloaded from the National Center for Biotechnology Information (NCBI) FTP web site ftp://ftp.ncbi.nlm.nih.gov/genomes in August 2015. These sequences were aligned using the *Parasail* software package [1, 2] and then clustered using *Grappolo* [3] based on their similarity scores. After completion of clustering, an R script was used to create an $n \times m$ matrix with n rows representing the number of protein clusters and m columns representing each organism. An entry of 1 in cell c_{ij} meant that the organism j had at least one protein sequence in the protein cluster i; an entry of 0 meant that it did not. This matrix provided the basis for computing the distances between the organisms where each organism was represented by its 0-1 column vector. The distances were computed using the L_1 distance given by:

$$d(p,q) = \|p - q\|_1 = \sum_{i=1}^{n} |p_i - q_i|$$

The L_1distance is well suited for discrete values. Essentially it measures the number of changes from 0 to 1 or from 1 to 0 to transform from one vector into another. After the distances were computed, *visone*, a software package available on the web for analysis and visualization of social networks, was used to construct the network [4]. In the following sections, we provide details for each computational step.

Protein sequence alignment with *Parasail*

Parasail is a SIMD C (C99) library containing implementations of the Smith-Waterman local alignment, Needleman-Wunsch global alignment, and semi-global pairwise sequence alignment algorithms [1]. Here, semi-global means insertions before the start or after the end of either the query or target sequence are not penalized. The three algorithms are guaranteed to find optimal alignments. *Parasail* implements vectorized versions of most known algorithms for pairwise sequence alignment and significantly speeds up their computation. For example, alignment of the full set of protein sequences for the 102 genomes, i.e., 120K sequences, took approximately 5 minutes on a desktop computer.

Table S2.1. **Default parameters used by *Parasail***.

Parameter Name	Parameter Value
Exact Match Length	7
Gap Extend Penalty	1
Gap Opening Penalty	10
Alignment Length, %	80%
Match Similarity, %	40%
Optimal Score, %	30%
Bit Precision	16
Scoring Matrix	blosum62

For our network, we used the semi-global pairwise sequence alignment algorithm as a compromise between the local and global algorithms. All parameters used in the algorithm were set to default (S2 Table). What follows is a brief description of these three important parameters.

Alignment Length (AL)

This parameter indicates the minimum required length of the alignment relative to the longer sequence. For example, if the longer sequence is 20 residues and AL=80%, then at least 16 residues have to be involved in the alignment region. The default value is 80%.

Match Similarity (MS)

This parameter measures the percent identity within the alignment. It is the percent similarity between sequences. The default value is 40% because two sequences are said to be “homologous” if they share a local alignment with a minimum 40% identity and if the alignment covers at least 80% of the longer sequence [5].

Optimal Score (OS)

The optimal score parameter is the ratio of the alignment score and the self-score. The optimal score is the better one of the two sequences. The default value is 30%.

AL, MS, and OS are used during alignment to test whether a sequence pair is connected by an edge. If all three parameters are satisfied, then the pair of protein sequences is connected by an edge. As its output, *Parasail* returns a graph and three alignment statistics for each edge computed. The statistics are length of alignment over maximum length, number of exact matches over alignment length, and alignment score over self-score. The edge information can be used to identify clusters of similar protein sequences. We used the first of these statistics as the input parameter for the clustering algorithm *Grappolo*.

Clustering with *Grappolo*

Grappolo implements parallelization of the Louvain heuristic for community detection in large-scale graphs [3]. In our application, a “community” is a set of closely related protein sequences. Thus, Grappolo clusters protein sequences based on their similarity measure as computed by Parasail (see above). Grappolo has been shown to produce clusters of high modularity [3]. By inspection of the clusters that were created, we observed their proteins to be closely related in sequence as well as in function (S3 Table). Also, because of its multi-threaded implementation using OpenMP, Grappolo is very fast. For our data there are approximately 2M edges connecting similar sequences. High-homogeneity protein clusters were identified by Grappolo in less than 1 minute.

The proposed pipeline of *pClust = Parasail + Grappolo* can be viewed as a computationally effective alternative for identification of groups of homologous genes. *Parasail* provides fast alignment of protein sequences while *Grappolo* executes their accurate clustering. The result is protein clusters of high sequence similarity.

Table S2.2. **Example of a protein cluster produced by *Grappolo***.

<table>
<tr><th>Cluster 67: 26 Sequences</th></tr>
<tr><td>76 >AAV86266.1 riboflavin synthase alpha chain [Anaplasma marginale str. St. Maries]</td></tr>
<tr><td>1701 >AAZ68804.1 Lumazine-binding protein [Ehrlichia canis str. Jake]</td></tr>
<tr><td>2751 >ABD44101.1 riboflavin synthase, alpha subunit [Anaplasma phagocytophilum str. HZ]</td></tr>
<tr><td>3542 >ABD44804.1 riboflavin synthase, alpha subunit [Ehrlichia chaffeensis str. Arkansas]</td></tr>
<tr><td>4406 >ACM48976.1 riboflavin synthase alpha chain (ribE) [Anaplasma marginale str. Florida]</td></tr>
<tr><td>6156 >ACZ49612.1 riboflavin synthase subunit alpha [Anaplasma centrale str. Israel]</td></tr>
<tr><td>6931 >CAH58471.1 putative riboflavin synthase, alpha subunit [Ehrlichia ruminantium str. Welgevonden]</td></tr>
<tr><td>7855 >CAI28222.1 Riboflavin synthase alpha chain [Ehrlichia ruminantium str. Gardel]</td></tr>
<tr><td>8814 >CAI27273.1 Riboflavin synthase alpha chain [Ehrlichia ruminantium str. Welgevonden]</td></tr>
<tr><td>9061 >AGR78604.1 riboflavin synthase subunit alpha [Anaplasma phagocytophilum str. HZ2]</td></tr>
<tr><td>10308 >AGR79851.1 riboflavin synthase subunit alpha [Anaplasma phagocytophilum str. JM]</td></tr>
<tr><td>12118 >AHC39487.1 riboflavin synthase subunit alpha [Ehrlichia muris AS145]</td></tr>
<tr><td>12590 >AHX03370.1 riboflavin synthase, alpha subunit [Ehrlichia chaffeensis str. Heartland]</td></tr>
<tr><td>13821 >AHX04601.1 riboflavin synthase, alpha subunit [Ehrlichia sp. HF]</td></tr>
<tr><td>15131 >AHX05911.1 riboflavin synthase, alpha subunit [Ehrlichia chaffeensis str. Jax]</td></tr>
<tr><td>16121 >AHX06901.1 riboflavin synthase, alpha subunit [Ehrlichia chaffeensis str. Liberty]</td></tr>
<tr><td>16508 >AHX07288.1 riboflavin synthase, alpha subunit [Ehrlichia chaffeensis str. Osceola]</td></tr>
<tr><td>17323 >AHX08103.1 riboflavin synthase, alpha subunit [Ehrlichia chaffeensis str. Saint Vincent]</td></tr>
<tr><td>18531 >AHX09311.1 riboflavin synthase, alpha subunit [Ehrlichia chaffeensis str. Wakulla]</td></tr>
<tr><td>19943 >AHX10723.1 riboflavin synthase, alpha subunit [Ehrlichia chaffeensis str. West Paces]</td></tr>
<tr><td>25508 >gi|49474162|ref|YP_032204.1| riboflavin synthase subunit alpha [Bartonella quintana str. Toulouse]</td></tr>
<tr><td>26873 >gi|49475528|ref|YP_033569.1| riboflavin synthase subunit alpha [Bartonella henselae str. Houston-1]</td></tr>
<tr><td>56437 >gi|163868465|ref|YP_001609674.1| riboflavin synthase subunit alpha [Bartonella tribocorum CIP 105476]</td></tr>
<tr><td>69195 >gi|240850689|ref|YP_002972089.1| riboflavin synthase subunit alpha [Bartonella grahamii as4aup]</td></tr>
<tr><td>70708 >gi|319898991|ref|YP_004159084.1| Riboflavin synthase alpha chain [Bartonella clarridgeiae 73]</td></tr>
<tr><td>111984 >gi|403530440|ref|YP_006664969.1| riboflavin synthase subunit alpha [Bartonella quintana RM-11]</td></tr>
</table>

Computing organism distance matrix

A 0-1 matrix is constructed after every protein sequence is assigned to a cluster. A part of such a matrix is shown in S4 Table. An entry of 1 in a cell means that the specific organism has at least one protein sequence in the given protein cluster; an entry of 0 means that it does not. The 0-1 vectors serve as a basis for computing pairwise distances between organisms using the L_1 distance (see above). For example, if *A. marginale* str. St. Maries and *E. canis* str. Jake had only the fourteen clusters shown in S4 Table, then the distance between these two strains would have been 6, i.e., the number of cells for which entries differ between the two strains. However, we discovered that distance itself did not provide sufficient resolution for network construction. In order to enhance the resolution, the calculated distance was first normalized by dividing each row entry by the row sum and then inverted by taking its reciprocal. The resulting distance matrix served as the foundation for network visualization with *visone*.

Table S2.3. **Portion of the 0-1 matrix.** An entry of 1 in a cell means that the specific organism has at least one protein sequence in the given protein cluster; an entry of 0 means that it does not.

Cluster	Anaplasma marginale str. St. Maries	Ehrlichia canis str. Jake	Anaplasma phagocytophilum str. HZ	Ehrlichia chaffeensis str. Arkansas	Anaplasma marginale str. Florida	Anaplasma centrale str. Israel
**Cluster 1**	1	0	0	0	1	1
**Cluster 2**	1	1	1	1	1	1
**Cluster 3**	1	0	1	0	1	1
**Cluster 4**	1	0	0	0	1	1
**Cluster 5**	1	1	1	1	1	1
**Cluster 6**	1	1	1	1	1	1
**Cluster 7**	1	0	0	0	1	1
**Cluster 8**	1	0	0	0	1	1
**Cluster 9**	1	1	1	1	1	1
**Cluster 10**	1	1	1	1	1	1
**Cluster 11**	1	1	1	1	1	1
**Cluster 12**	1	1	1	1	1	1
**Cluster 13**	1	0	0	0	1	0
**Cluster 14**	1	1	1	1	1	1

Visualization with *visone*

Visualization was done using *visone* v. 2.10 [4], a program free for nonprofit use. Originally *visone* was designed specifically for a class of small-world graphs, but we found that it provided accurate depictions of our organism networks. This suggests that microorganisms in our set have the same properties as small-world graphs; namely, they have high density, low diameter, and noisy group structure. The method is based on a spanning subgraph that is sparse but connected and consists of strong ties holding together communities. For the actual drawing of the network several options are available for the backbone layout. In the baseline comparison study (see below) we did not insist on keeping our network connected, retaining only the top 20% of the strongest connections. For our network of 102 organisms (Fig. 1), we kept the network connected and retained all the connections with values above the mean. The difference in approaches is largely dictated by the size of the sets and the fact that in the baseline

comparison study we used only non-singleton clusters. For larger organism sets, the edge filtering above the mean value seems to provide a better differentiation among closely related organisms such as, for example, the *Rickettsia* cluster (Fig. 1).

Baseline comparison study for network construction

In order to verify that our method creates accurate biological networks, we first constructed a network for a smaller set of eleven organisms to compare with a well-known phylogenomic structure obtained by Gillespie *et al.* [6]. Ten of the eleven organisms are members of the *Rickettsial* group; one organism, *Wolbachia endosymbiont*, served as an out-of-set member for tree construction (S5 Table). Observe that when using our phylogenomic network construction approach, there is no need for an out-of-set member, but we included *Wolbachia endosymbiont* both for consistency in comparing with the results of Gillespie *et al*. and to confirm it as an outlier. In total the eleven organisms contained 13571 protein sequences that were aligned with *Parasail* and grouped with *Grappolo* as described above.

Table S2.4. **The 11 organisms used for the baseline comparison study**. *Rickettsia* four major group abbreviations – ancestral group (AG), typhus group (TG), transitional group (TRG), and spotted fever group (SFG).

Organism Name	Vector	Group	Protein Sequences File	Proteins
Rickettsia akari str. Hartford	Mites	TRG	NC_009881.1	1257
Rickettsia felis URRWXCal2	Fleas	TRG	NC_007109.1	1400
Rickettsia prowazekii str. NMRC Madrid E	Lice, Fleas	TG	NC_020992.1	938
Rickettsia typhi str. Wilmington	Fleas	TG	NC_006142.1	837
Rickettsia conorii str. Malish 7	Tick	SFG	NC_003103.1	1374
Rickettsia rickettsii str. 'Sheila Smith'	Tick	SFG	NC_009882.1	1343
Rickettsia sibirica 246	Tick	SFG	NZ_AABW01000001.1	1234
Rickettsia bellii OSU 85-389	Tick	AG	NC_009883.1	1475
Rickettsia bellii RML369-C	Tick	AG	NC_007940.1	1429
Rickettsia canadensis str. McKiel	Tick	AG	NC_009879.1	1089
Wolbachia endosymbiont	---	---	NC_002978.6	1195

The phylogenomic tree relating the ten organisms in [6] was produced using alignment of 731 representative core Rickettsial proteins defined as orthologous proteins with only one ORF per included genome [6] which were identified by the OrthoML program [7]. To create similar settings for our network construction we utilized only non-singleton protein clusters. A *singleton protein cluster* is a "cluster"[1] containing only one sequence. These sequences represent protein products unique to a specific organism. In contrast, a *non-singleton (NS) protein cluster* is a cluster with at least two protein sequences. It may be the case that sequences in an NS cluster come from the same organism, but in most NS clusters the sequences are distributed among a number of organisms. NS clusters can be viewed as an alternative to groups of homologous proteins. An example of an NS cluster can be seen in S3 Table.

[1] The word "cluster" is put in double quotes because *cluster* implies a collection of items of more than one. However, a *singleton cluster* has exactly one protein sequence in it.

The left side of Fig. S4 reproduces the tree from [6] constructed for the 10 *Rickettsia* organisms with *Wolbachia* as an outlier. The study by Gillespie *et al.* classified these members of *Rickettsia* into four major groups – ancestral group (AG), typhus group (TG), transitional group (TRG), and spotted fever group (SFG). The tree was constructed using Bayesian analysis in which chains were primed using a neighbor-joining tree and run independently for 25,000 generations in model-jumping mode (Fig. S4). This tree is almost identical to another tree which was obtained from exhaustive search using parsimony, which was feasible to create because only 11 organisms were involved (including *Wolbachia*). See ref. 6 for details.

Figure S2.1. ***Rickettsia* tree (left) versus *Rickettsia* network (right).** The tree is reproduced from the work of Gillespie *et al*. Fig.4A [6]. AG – ancestral group, TG – typhus group, TRG – transitional group, SFG – spotted fever group. Edge thickness indicates organism relatedness, i.e., the thicker the edge, the more closely related the organisms are.

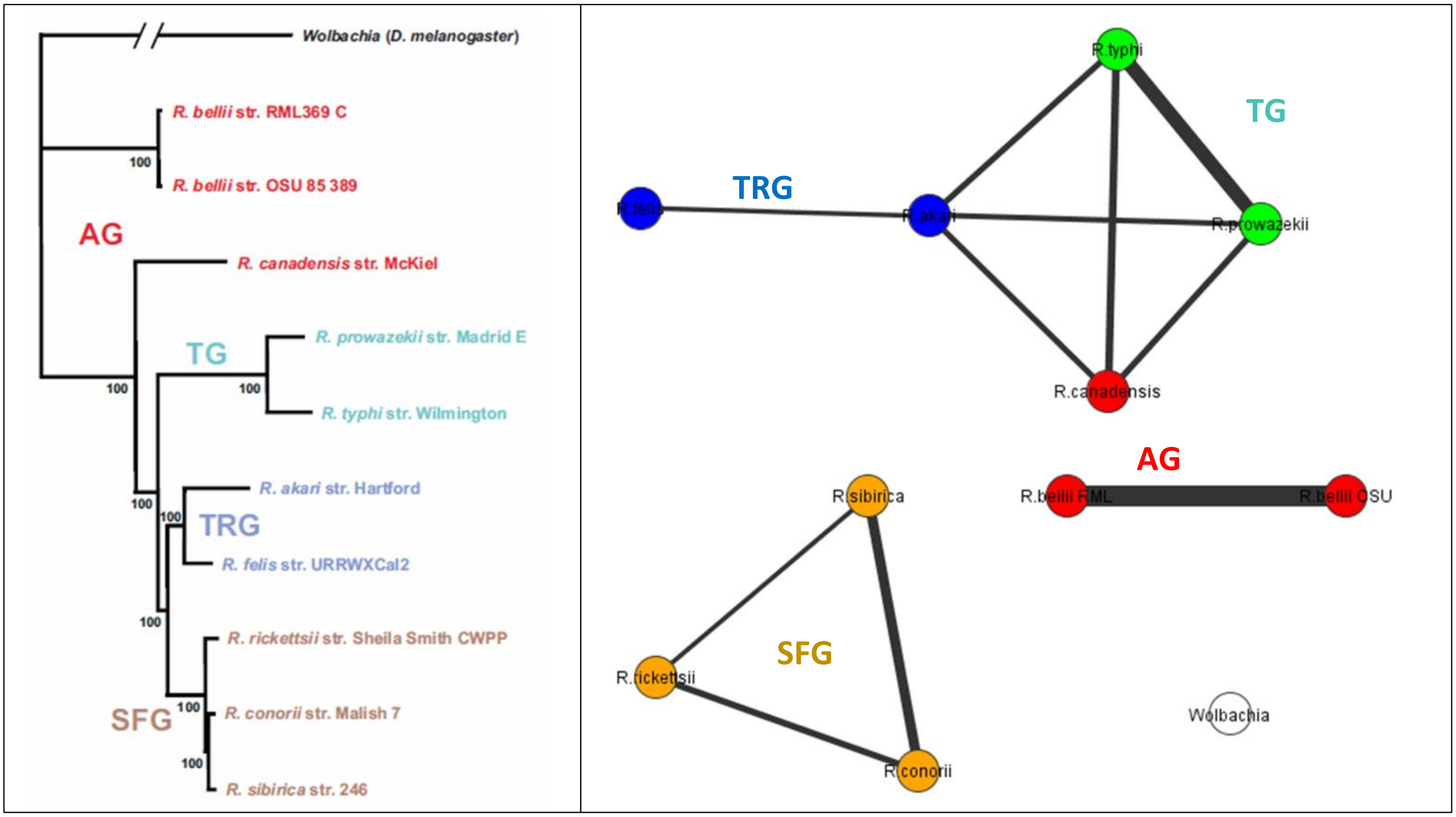

In Fig. 1, lines (edges) connecting organisms are of equal thickness because of the complexity of the network. However, for smaller networks line thickness can be used to represent greater similarity between organisms as demonstrated in Fig. S4. The right side of Fig. S4 shows the phylogenomic network constructed using our method for the same set of organisms studied by Gillespie. Only NS clusters were used, the total number of which were 1687 for the ten *Rickettsial* organisms. We can see the important similarities between the two structures in Fig. S4. In particular, *Wolbachia*'s position confirms it as an outlier in this set. The members of the spotted fever group (SFG) – *R. sibirica, R. conorii*, and *R. rickettsii* – form a triangle with *R. sibirica* and *R. conorii* which are joined by a thicker edge

indicating their greater similarity. The members of TRG, *R. felis* and *R. akari*, are connected as well as are *R. typhi* and *R. prowazekii* (members of TG). The two *R. bellii* belonging to the ancestral group are also connected together by a very thick edge. *R. canadensis* is in an interesting position. While it is classified as a member of the AG, it is more closely related to members of the typhus group than to the *R. bellii* members of the ancestral group. This fact is reflected both in the tree and in the network (Fig. S4). In the tree, *R. canadensis* is on the same branch-off from *R. bellii* as TG and TRG while in the network it has connections with both members of TG and a connection to TRG and none with AG. In either case, such positioning of *R. canadensis* suggests that evolutionary distance from *R. canadensis* to TG members is much closer than to AG members. Another interesting difference between the tree and the network is that the members of TG and TRG and *R. canadensis* form a connected cluster. This could be because inherently information reflected in networks is more complex than what can be displayed in trees, as networks allow interconnectedness among nodes. Thus, the exact positions and connections among the nodes of trees and networks are different by the very virtue of their construction. However, such clustering may also suggest that evolutionary distances among these organisms are much smaller than from AG and SFG members. This point, though, needs further investigation.

Overall, our network construction method appears to accurately capture the relationships among bacterial organisms. It delivers consistent results with existing phylogeny while at the same time providing new insights. Importantly, the method is also computationally very fast and suitable for desktop use. The tree obtained by Gillespie *et al.* using Bayesian analysis for 11 genomes required parallel computation on a large cluster whereas the network obtained for the 102 complete genomes shown in Fig. 1 required less than 10 minutes on a desktop computer.

Software Availability

The open-source GUI software package *pClust* and custom R scripts were used for phylogenomic network construction. *pClust* and R scripts are available for download at https://bitbucket.org/wsu_bcb/pClust. *pClust* utilizes the *Parasail* package for fast sequence alignment and *Grappolo* for clustering. *Parasail* and *Grappolo* are open source software packages. Their source code is available for download, *Parasail* at https://github.com/jeffdaily/parasail and *Grappolo* at http://hpc.pnl.gov/people/hala/grappolo.html. Network visualization was done using the free software package *visone* downloadable at http://visone.info.

References

1. Daily J. Scalable Parallel Methods for Analyzing Metagenomic Data at Extreme Scale [Doctor of Phylosophy]: Washington State University; 2015.
2. Daily J, Kalyanaraman A, Krishnamoorthy S, Vishnu A. A work stealing based approach for enabling scalable optimal sequence homology detection. J Parallel Distr Com 2015;79-80:132-42.
3. Lu H, Halappanavar M, Kalyanaraman A. Parallel heuristics for scalable community detection. Parallel Com. 2015;47:19-37.
4. Nocaj A, Ortmann M, Brandes U. Untangling the Hairballs of Multi-Centered, Small-World Online Social Media Networks. J Graph Algorithms and Applications. 2015;19(2):595-618.
5. Wu C, Kalyanaraman A, Cannon WR. pGraph: Efficient Parallel Construction of Large-Scale Protein Sequence Homology Graphs. IEEE Trans Parall Distr Sys. 2012;23(10):1923-33.

6. Gillespie JJ, Williams K, Shukla M, Snyder EE, Nordberg EK, Ceraul SM, et al. Rickettsia phylogenomics: unwinding the intricacies of obligate intracellular life. PLoS One. 2008;3(4):e2018.
7. Li L, Stoeckert CJ, Jr., Roos DS. OrthoMCL: identification of ortholog groups for eukaryotic genomes. Genome Res. 2003;13(9):2178-89.
