# Supplementary material for: Comparative genomics reveals multiple pathways to mutualism for tick-borne pathogens
Source: BMC Genomics. 2016 Jul 2;17:481. doi: 10.1186/s12864-016-2744-9 (PMC4930560; doi:10.1186/s12864-016-2744-9)

Figure S1. **Three-dimensional depiction of multiplicity of gene clusters according to vector species**. Every ball represents a gene cluster; its location in (x, y, z) coordinates gives the percentage proportional to its appearance in the genome of an organism that is transmitted by one of the three vector types – ticks, lice, and fleas, respectively. The size of balls corresponds to the multiplicity of protein clusters, i.e., larger balls correspond to frequently appearing protein clusters.

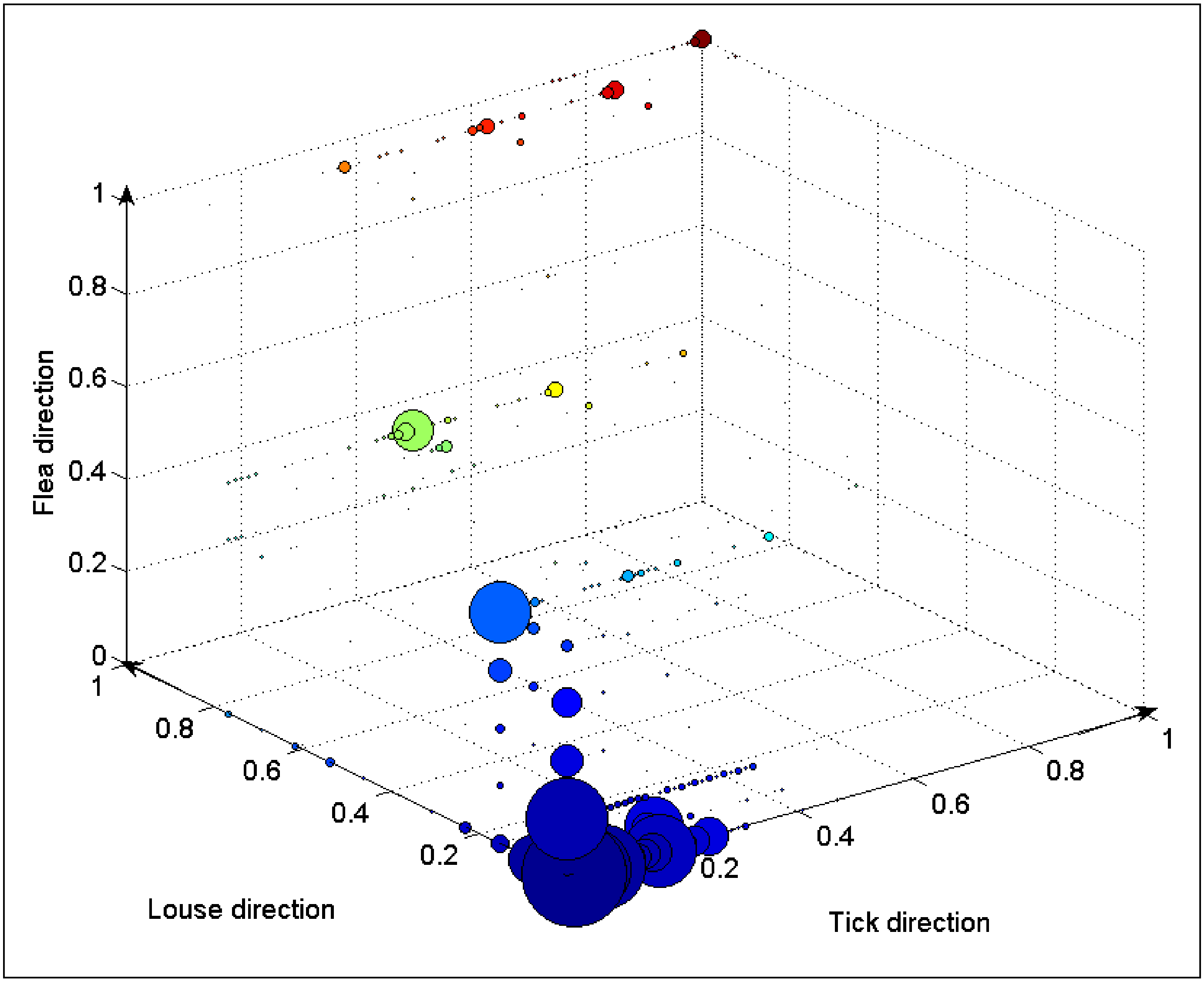

Figure S2. **The span of three gene clusters across different tick-borne organisms.** Black color where a homologous gene is present, white – absent. The KEGG annotations of the enzymes corresponding to the gene clusters are along the vertical axis. They are: EC 5.4.2.12 – 2,3-bisphosphoglycerate-independent phosphoglycerol mutase; EC 2.4.2.19 – nicotinate-nucleotide pyrophosphorylase; EC 2.5.1.72 – quinolinate synthetase A. The species are located along the horizontal axis at the following positions: *Anaplasma* spp. – 1-6, *Ehrlichia* spp. – 7-20, *Francisella* spp. – 21-33, *Rickettsia* spp. – 34-59, *Borrelia* spp. – 60-76, *Coxiella* spp. – 77-81.

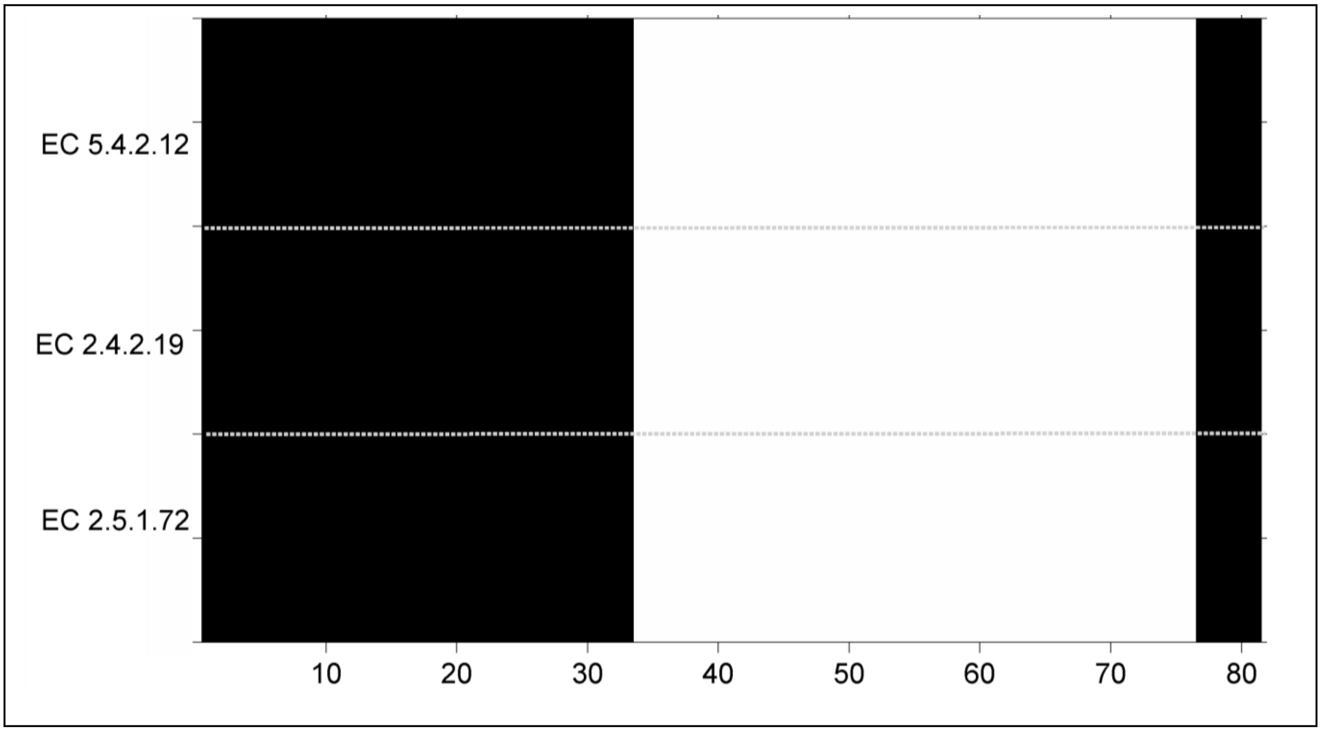

Figure S3. **The span of two gene clusters across different tick-borne organisms.** Black color where a homologous gene is present, white – absent. The KEGG annotations of the enzymes corresponding to the gene clusters are along the vertical axis. They are: EC 1.1.1.267 – 1-deoxy-D-xylulose-5-phosphate reductoisomerase; EC 4.6.1.12 – 2-C-methyl-D-erythritol 2,4-cyclodiphosphate synthase. The species are located along the horizontal axis at the following positions: *Anaplasma* spp. – 1-6, *Ehrlichia* spp. – 7-20, *Francisella* spp. – 21-33, *Rickettsia* spp. – 34-59, *Borrelia* spp. – 60-76, *Coxiella* spp. – 77-81.

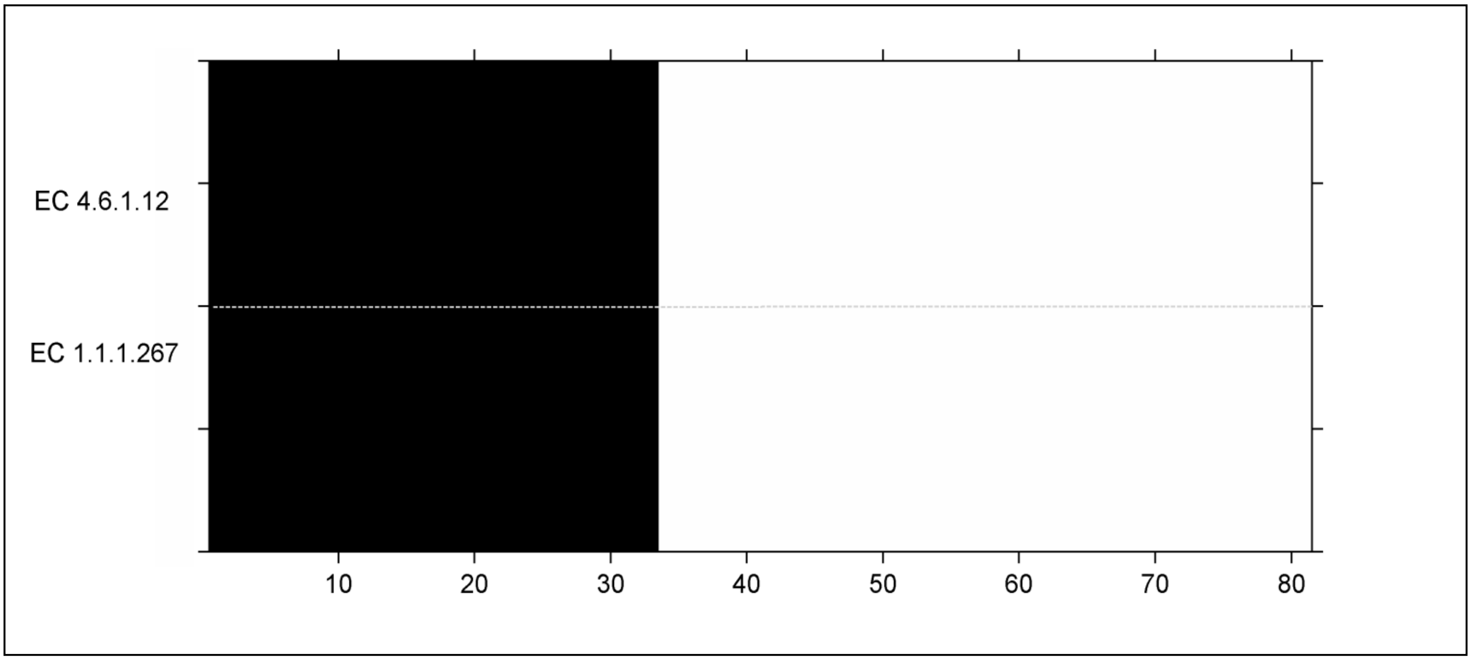

Supplement: Additional file 3 — Figure S2. The span of three gene clusters across different tick-borne organisms. Figure S3. The span of two gene clusters across different tick-borne organisms. (PDF 606 kb) [file 12864_2016_2744_MOESM3_ESM.pdf]
